# A Proteomic Approach to Understand the Clinical Significance of Acute Myeloid Leukemia–Derived Extracellular Vesicles Reflecting Essential Characteristics of Leukemia

**DOI:** 10.1074/mcp.RA120.002169

**Published:** 2020-12-08

**Authors:** Ka-Won Kang, Hyoseon Kim, Woojune Hur, Jik-han Jung, Su Jin Jeong, Hyunku Shin, Dongkwon Seo, Hyesun Jeong, ByeongHyeon Choi, Sunghoi Hong, Hyun Koo Kim, Yeonho Choi, Ji-ho Park, Kil Yeon Lee, Kwang Pyo Kim, Yong Park

**Affiliations:** 1 Division of Hematology-Oncology, Department of Internal Medicine, Korea University College of Medicine, Seoul, South Korea; 2 Department of Applied Chemistry, Institute of Natural Science, Global Center for Pharmaceutical Ingredient Materials, Kyung Hee University, Yongin, South Korea; 3 Department of Biomedical Science and Technology, Kyung Hee Medical Science Research Institute, Kyung Hee University, Seoul, South Korea; 4 Department of Bio and Brain Bioengineering, Korea Advanced Institute of Science and Technology (KAIST), Daejeon, South Korea; 5 Department of Statistics Support, Medical Science Research Institute, Kyung Hee University Hospital, Seoul, South Korea; 6 Department of Bio-convergence Engineering, Korea University, Seoul, South Korea; 7 School of Biosystem and Biomedical Science, Korea University, Seoul, South Korea; 8 Department of Thoracic and Cardiovascular Surgery, Korea University College of Medicine, Seoul, South Korea; 9 Department of Surgery, College of Medicine, Kyung Hee University, Seoul, Republic of Korea

**Keywords:** Acute myeloid leukemia, Extracellular vesicle, Proteomics, LC-MS, ACN, acetonitrile, AML, acute myeloid leukemia, BM, bone marrow, CI, confidence interval, CR, complete remission, DEPs, differentially expressed proteins, DLS, dynamic light scattering, EV, extracellular vesicle, FBS, fetal bovine serum, FC, fold change, FDR, false discovery rate, FPKM, fragments per kilobase of transcript per million, HDFa, human dermal fibroblasts, adult, hMSCs, human mesenchymal stem cells, HR, hazard ratio, LC-MS/MS, liquid chromatography–tandem mass spectrometry, RFS, relapse-free survival, TCGA, The Cancer Genome Atlas, TEM, transmission electron microscopy, TMT, tandem mass tag

## Abstract

Extracellular vesicle (EV) proteins from acute myeloid leukemia (AML) cell lines were analyzed using mass spectrometry. The analyses identified 2450 proteins, including 461 differentially expressed proteins (290 upregulated and 171 downregulated). CD53 and CD47 were upregulated and were selected as candidate biomarkers. The association between survival of patients with AML and the expression levels of CD53 and CD47 at diagnosis was analyzed using mRNA expression data from The Cancer Genome Atlas database. Patients with higher expression levels showed significantly inferior survival than those with lower expression levels. ELISA results of the expression levels of CD53 and CD47 from EVs in the bone marrow of patients with AML at diagnosis and at the time of complete remission with induction chemotherapy revealed that patients with downregulated CD53 and CD47 expression appeared to relapse less frequently. Network model analysis of EV proteins revealed several upregulated kinases, including LYN, CSNK2A1, SYK, CSK, and PTK2B. The potential cytotoxicity of several clinically applicable drugs that inhibit these kinases was tested in AML cell lines. The drugs lowered the viability of AML cells. The collective data suggest that AML cell–derived EVs could reflect essential leukemia biology.

## Highlights

• AML-derived EVs are key cargo proteins carrying parental AML cell information.
• Proteomics of AML-derived EVs identify biomarkers predicting therapeutic responses.
• EVs' CD53 and CD47 expression levels may be related to the prognosis of AML patients.

## Graphical Abstract

**Figure:**
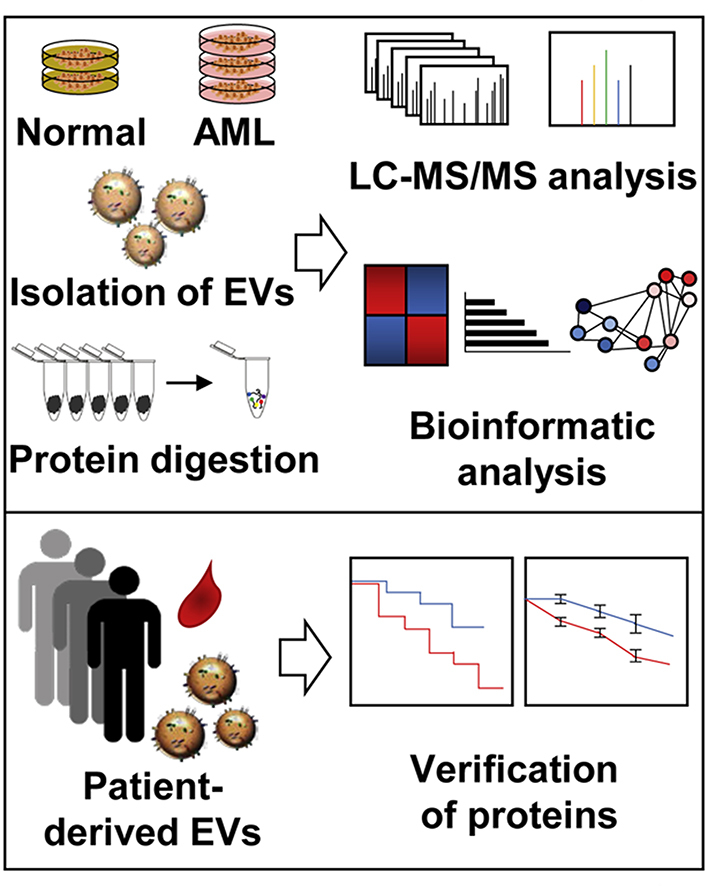

## In Brief

We analyzed proteomics of AML cell–derived extracellular vesicles (EVs) that carry key information of parental cells. CD53 and CD47 were selected as predictive EV markers for AML, which showed good correlation with relapse-free survival of patients. In addition, several tyrosine kinases were enriched in AML cell–derived EVs, which may serve as potential therapeutic targets for AML.

Acute myeloid leukemia (AML) is the most common form of acute leukemia in adults. The overall incidence of AML has been increasing gradually over the years (1, 2, 3). Despite many recent advances, the 7 + 3 chemotherapy regimen of cytarabine and anthracycline, originally developed in 1973, remains the basis of remission induction therapy. Morphologic analysis of marrow cells is still the gold standard for clinical diagnosis and response evaluation. The definition of complete remission (CR) for AML is based on the reduction of bone marrow (BM) blasts to 5% as determined by morphology (4). However, a considerable number of patients still experience relapse despite achieving morphological CR (5, 6). To overcome this drawback, various techniques have been developed to detect minimal residual disease. These techniques typically include PCR, flow cytometry, or next-generation sequencing (7, 8). However, there are still several limitations in the widespread use of these techniques for clinical practice. For example, flow cytometry requires specific and robust expertise to identify leukemia-associated immunophenotypes and uses an approach different from the normal one. Incorporating this type of analysis in clinical practice is also limited by the large amount of data that need to be dynamically evaluated to detect and analyze immunophenotype switches. In addition, the quality of the BM samples is imperative to all these analyses. If there is insufficient starting material, flow cytometry cannot be performed (7, 9).

Extracellular vesicles (EVs) express the properties of their parental cells, including proteins, RNAs, and DNAs (10). Cancer cell–derived EVs are involved in the overall etiology, including supporting tumor growth (11), inducing vessel formation (12) that contributes to the metabolic reprogramming of cancer cells, enabling sustained proliferation of cancer cells (13), and enhancing the capacity of tumors to become invasive (14, 15). Considering EVs are representative of their parental tissues, they are excellent tools to understand how cancer cells adapt to their environment. This has led to the recent emergence of EVs as a focus in cancer research. Clinically, several studies have documented the potential uses of EVs in treating patients with cancer. One study reported that circulating EVs that were glypican-1–positive could be used as biomarkers for early detection and prognosis in patients with pancreatic cancer (16). Another study reported that EVs from drug-resistant breast cancer cells could be used as therapeutic targets to enhance patients' therapeutic responses (17).

The use of EVs as biomarkers of AML has been studied. The plasma levels of exosomes in patients newly diagnosed with AML (expressed as μg/protein/ml) were higher than those in normal controls (18). Plasma exosome levels of EVs were reduced after a course of remission induction therapy concomitant with the reduction of blasts in BM (19). A study of exosomes derived from primary AML cells and AML cell lines showed that these exosomes contained coding and noncoding RNAs relevant to AML pathogenesis that affected prognosis, response to therapy, and leukemic niche formation (20). Another study using a leukemic xenograft mouse model showed that miRNAs in AML exosomes could serve as early biomarkers of relapse (21). In addition, proteomics analyses have demonstrated the involvement of AML-derived exosomes in leukemic transformation (22) and apoptosis inhibition (23).

Proteomic analysis is a useful technique in cancer research that can be both quantitative and qualitative when determining the inter-relationships between proteins in cells. Proteomics analyzes the phenotype of any expressed protein, and it can identify the type of protein expressed under varying conditions. This approach can also be used to identify potential disease-specific biomarker candidates (24). The composition of proteins in the blood of patients with leukemia is different from that of healthy individuals. These changes can interfere with the activities of healthy blood cells in affected patients (25, 26). Therefore, the types of proteins that make up the blood of healthy individuals and patients with leukemia as well as the respective signaling molecules and networks may be different. In particular, circulating EVs are characterized by signal cascades that can promote cancer metastases and their migration to other organs and tissues (27). Thus, it is clinically important to identify novel differentially expressed proteins (DEPs) from EVs in AML cells that could be used as biomarkers (28). In this study, we validated the use of proteomic analysis in cancer-derived EVs to identify and investigate potential biomarkers and novel drug targets for AML.

## Experimental Procedures

### Ethical Approval and Consent to Participate

All procedures for the culture of primary human BM stromal cells and acquisition of BM blood samples and patients' medical records were approved by the internal review board of the Korea University Anam Hospital (IRB No. 2015AN0267). Informed consent was obtained from all participants.

### Experimental Design and Statistical Rationale

This study was designed to perform proteomic analysis of EVs isolated from AML and control cell lines. For liquid chromatography–tandem mass spectrometry (LC-MS/MS) analysis, human dermal fibroblasts, adult (HDFa) and human mesenchymal stem cells (hMSCs) were analyzed as biological replicates in the control group. HL-60, KG-1, and THP-1 cells were analyzed as biological replicates in the AML group. Three technical replicates were performed on EVs derived from five cell lines. This sample size was statistically evaluated in the EV group derived from five types of cells, and the quantifiable protein was analyzed and compared in the AML and control groups. EVs were isolated from each cell culture supernatant via size-exclusion chromatography. Trypsin digestion of the isolated EV into a peptide was performed simultaneously with the same reagent to prevent variations in sample preparation, followed by analysis using the EASY-nLC 1000 system (Thermo Fisher Scientific, Bremen, Germany) connected to a Q-Exactive Orbitrap Hybrid Mass Spectrometer (Thermo Fisher Scientific, Bremen, Germany). The acquired MS/MS data were processed for protein identification and to search for quantitative information using Proteome Discoverer 2.4 software (Thermo Fisher Scientific, Bremen, Germany). Quantitative differences in the amount of peptides identified in EVs derived from the five cell lines were normalized as the mean of the total intensities of the peptide ion peaks obtained at three technical replicates for each sample. Statistical analysis was performed using IBM SPSS, version 25.0, software (IBM Corp). According to the normality result of the Shapiro–Wilk test, the parametric continuous data were statistically analyzed using one-way ANOVA test with the *p*-value, followed by the Bonferroni multiple comparison post hoc test to compare the differences in protein expression in the AML and control groups. To analyze the correlation between selected AML biomarkers and survival, information on 187 patients with AML was obtained from The Cancer Genome Atlas (TCGA) public database. To validate the selected AML biomarkers, BM serum or plasma samples from a total of 17 patients with AML were collected at two different time points. The statistical analysis of the experiments conducted in this work is described in more detail in the Statistical Analysis of Correlation of Survival Rates and AML-Derived EV Markers subsection.

### Cell Lines

HL-60, KG-1, THP-1, Kasumi-1, and MOLM-13 AML cell lines were selected for the study. HDFa and hMSCs were used as controls. HL-60, KG-1, THP-1, and Kasumi-1 cell lines were purchased from the American Type Culture Collection. The MOLM-13 cell line was purchased from the Japanese Collection of Research Bioresources. hMSCs were obtained from primary cultures of human BM. HL-60 and KG-1 cells were cultured in Iscove's Modified Dulbecco's Medium (Gibco). THP-1, Kasumi-1, and MOLM-13 cells were cultured in RPMI-1640 medium (Gibco). HDFa was cultured in Dulbecco's Modified Eagle's Medium (Gibco). hMSCs were cultured in the Mesenchymal Stem Cell Growth Medium (Lonza). All media were supplemented with 10% exosome-depleted fetal bovine serum (FBS; Gibco) and 1% penicillin/streptomycin (Gibco). Exosome-depleted FBS was prepared by collecting the supernatant after ultracentrifugation of normal FBS at 100,000*g*.

### Preparation of hMSCs and BM Samples

BM blood was collected from donations by healthy individuals for the BM transplantation treatment of recipients. BM blood (20 ml) was collected from each subject. Mononuclear cells were separated using Ficoll-Paque Plus (GE Healthcare Life Sciences) according to the manufacturer's instructions. BM serum or plasma samples from a total of 17 patients with AML were collected at the time of diagnosis and at the end of remission induction treatment. All patients achieved CR after anthracycline-based remission induction chemotherapy. The baseline characteristics of these patients are shown in Table 1. All human sample collections were performed according to the guidelines of the Internal Review Board of the Korean University Anam Hospital. Informed consent was obtained from all participants.

**Table 1:** Baseline characteristics of patients with acute myeloid leukemia

Baseline characteristics	Total patients (*n* = 17)
Median age, years (range)	53 (17–68)
Sex (male) (%)	9 (52.9)
Induction chemotherapy, *n* (%)	
Idarubicin based	13 (76.5)
Daunorubicin based	4 (23.5)
Cytogenetic risk, *n* (%)	
Favorable risk	7 (41.2)
Intermediate risk	8 (47.1)
Poor risk	2 (11.8)
Transplantation done, *n* (%)	12 (70.6)
Autologous bone marrow transplant	3 (17.6)
Allogeneic bone marrow transplant	9 (52.9)

### Isolation of EVs

Isolation of EVs was performed using size-exclusion chromatography. Different columns were used for cell culture supernatants and human samples. The column used for the cell culture supernatants was packed with 10-ml Sepharose CL-2B (GE Healthcare Life Sciences) with a molecular weight separation range of 70 × 10^3^–40 × 10^6^ (29). The eluted fractions (6, 7, 8, 9, and 10; 0.5 ml each) were used in the subsequent steps of this experiment. The loaded samples were prepared by sequential centrifugation. The cell culture media were collected after 48 to 72 h of cell growth (10 × 10^6^ cells in 50 ml) and centrifuged at 500*g* for 10 min at 4 °C and then at 5000*g* for 30 min at 4 °C, and finally at 10,000*g* for 30 min at 4 °C. The supernatant was concentrated using an Amicon Ultra 100-kDa filter with a molecular weight cut-off of 100 kDa (Merck Millipore) according to the manufacturer's instructions. For the human samples, EVs were isolated using a chromatography-based method developed by our group (30).

### EV Sizing and Evaluation Using Transmission Electron Microscopy and Dynamic Light Scattering (DLS)

Transmission electron microscopy (TEM) was performed using a model H-7500 transmission electron microscope (Hitachi). Dynamic light scattering (DLS) was performed using a Zetasizer Nano S90 (Malvern). For TEM analysis, EVs were fixed using 2% paraformaldehyde, loaded on a 300-mesh formvar/carbon-coated electron microscopy grid (Electron Microscopy Sciences), and stained with 2% phosphotungstic acid. For the DLS measurements, size distribution data were collected from each sample suspended in PBS. Each EV population was measured three times.

### Protein Extraction and Western Blotting

To extract protein, cell/EV pellets were lysed using ProEX CETi lysis buffer (Translab) followed by sonication. After sonication, nuclei and cell/EV membranes were separated by centrifugation at 10,000*g* for 15 min at 4 °C. The protein concentration was determined using the bicinchoninic acid protein assay kit (Pierce). In total, 30 μg of each protein sample was separated using 10% SDS-PAGE. The resolved proteins were transferred onto a nitrocellulose membrane. After blocking with the ProNA General-BLOCK solution (Translab) for 1 h, the membranes were probed overnight at 4 °C with 1:1000 dilutions of mouse anti-CD4 mAb, mouse anti-CD9 mAb, mouse anti-CD33 mAb, mouse anti-CD47 mAb, mouse anti-CD53 mAb (all from Santa Cruz Biotechnology), rabbit polyclonal anti-CD81 antibody (Bioss Antibodies), and rabbit anti-calnexin mAb (Cell Signaling Technology) as primary antibodies. Peroxidase-conjugated anti-mouse or anti-rabbit antibody (1:2000; Santa Cruz Biotechnology) was used as the secondary antibody. The antibody–antigen reactions were visualized using the ProNA ECL Ottimo kit (Translab), and the images were acquired using the ChemiDoc Touch imaging system (Bio-Rad).

### Tryptic Digestion by Filter-Aided Sample Preparation

In total, 30 μg of protein from EVs of AML or control cells was converted to peptides using the filter-aided sample preparation method. Extracted EV proteins were resolved by reduction in 0.1 M DTT and 4% SDS and were reconstituted in 0.1 M Tris-HCL (pH 7.6). Samples were incubated for 45 min at 37 °C and then boiled for 7 min. Samples were then centrifuged at 14,000*g* for 40 min at 16 °C using a 30-kDa Microcon Ultracel filter (Millipore), washed with 200 μl of the urea buffer (8 M urea in 0.1 M Tris-HCl, pH 8.5), and centrifuged three times. The proteins were then alkylated in urea buffer with 100 μl of 55-mM indoleacetic acid, incubated at RT for 20 min, and centrifuged for 40 min. The samples were then washed three times. Filters were placed in a 1.5-mL tube for collection. Digestion was performed using trypsin in 100-mM triethylammonium bicarbonate (TEAB) for 12 h at 37 °C. Peptides were harvested by centrifugation at 14,000*g* for 20 min at 16 °C, followed by the addition of 75 μl of 100-mM TEAB for two additional harvests. Harvested peptides were dried and resolved in 180 μl of 5% acetonitrile (ACN) with 0.1% formic acid. Samples were desalted using a C-18 Spin Column (Thermo Fisher Scientific) and dried in a vacuum concentrator (Hanil Scientific Inc).

### Tandem Mass Tag Labeling

Up to 0.8 mg of the tandem mass tag (TMT)-126, TMT-127, TMT-128, TMT-129, and TMT-130 reagents were resolved using 41 μl of anhydrous ACN. Each peptide sample was labeled by mixing it with 41 μl of the corresponding TMT reagent and incubating for 1 h. Eight microliters of 5% hydroxylamine was added to each sample and incubated for 15 min to allow quenching. Each sample was labeled with TMT 5plex according to the manufacturer's instructions (Thermo Fisher Scientific, Bremen, Germany): HDFa (126), hMSCs (127), HL-60 (128), KG-1 (129), and THP-1 (130).

### High-pH Fractionation

To increase the number of peptides identified in each sample, high-pH fractionation was used to separate peptides based on hydrophobicity. Samples were separated into 48 fractions using an XBridge BEH C18 column (130 Å, 5 μm, 4.6 mm × 250 mm; Waters Corp) on a 1100 series HPLC system (Agilent Technologies). The column was equilibrated in buffer A (10-mM ammonium formate in water, pH 10.0) before sample injection. The samples were fractionated under a linear gradient of 0%–70% buffer B (10-mM ammonium formate in 90% ACN, pH 10) at a flow rate of 500 μl/min for 115 min. The gradient conditions were as follows: 0 to 10 min, 5% B; 10 to 20 min, 5% B; 20 to 80 min, 35% B; 80 to 95 min, 70% B; 95 to 105 min, 70% B; and 105 to 115 min, 0% B. The fractions were dried in a SpeedVac device. Fractionated samples were desalted using a C18 spin column as described previously (31).

### Protein Identification by MS

Fractionated peptide samples were resolved in 0.1% formic acid and analyzed using an EASY-nLC 1000 system connected to a Q-Exactive Orbitrap Hybrid Mass Spectrometer. For the proteome profiling analysis, the sample was analyzed using a linear gradient of buffer A (solvent A: 0.1% formic acid in water) and solvent B (0.1% formic acid in ACN). The gradient applied was as follows: 0 to 5 min, 5% B, 5 to 105 min, 40% B, 105 to 110 min, 80% B, 110 to 115 min, 80% B, 115 to 116 min, 5% B, and 116 to 120 min, 5% B. The peptides eluted through the trap were ionized on an EASY-spray column (50 cm × 75 μm i.d.; Thermo Fisher Scientific, Bremen, Germany) packed with 2 μm C-18 particles at an electrospray voltage of 1.8 kV. Full MS data were acquired using positive polarity, a scan range of 400 to 2000 m/z, resolution of 70,000, automatic gain control target value of 1.0 × 10^6^, and a maximum ion-injection time of 100 ms. The maximum ion-injection time for MS/MS was set to 150 ms at a resolution of 17,500 in the centroid mode, with a maximum target capacity of the C-trap (automatic gain control target) of 1.0 × 10^6^. The dynamic exclusion time was set to 30 s, and the normalized higher collisional dissociation collision energy was set to 30. The raw LC-MS/MS data are available at www.proteomexchange.org under the data set identifier number PXD022758.

### Raw-Data Processing

The MS/MS spectra were analyzed using the SEQUEST HT search engine (Thermo Fisher Scientific, Bremen, Germany) and Proteome Discoverer 2.4 software (Thermo Fisher Scientific) to search the UniProtKB human protein database (UniProtKB/SwissProt, reviewed in June 2020, with 20,370 entries), which generated a decoy spectrum library of all human databases. Protein Discoverer 2.4 parameters set up cleavage at arginine and lysine residues using trypsin. Two missed cleavages were allowed. For each sample search, the carbamidomethylation of cysteine, TMT tag on lysine, and the N-terminus were set as a static modification, and N-acetylation and oxidation of methionine as variable modifications. A 1% false discovery rate (FDR) threshold was applied to the target peptide spectrum match and protein level analyses. The tolerance was set to 30 ppm for the precursor masses and 0.02 Da for the fragment masses. The threshold score for peptide identification was equivalent to 1% FDR. The FDR was calculated by searching the aforementioned decoy human protein sequence database produced by Protein Discoverer 2.4 against the UniProtKB human protein database. Semiquantitative analysis was performed via total spectral counts for proteins identified using the Protein Discoverer software. Only proteins quantified and identified with at least one unique peptide (clearly assigned to the peptide belonging to the protein, as evaluated by the Protein Discoverer 2.4 software) were subjected to statistical analysis. The information on assigned peptide sequences and protein identifications was acquired via ProteomeXchange with an identifier, PXD022758 based on UniProtKB/SwissProt database. The information of protein and peptide identifications such as accession numbers, the number of peptides for each protein, and the percentage of coverage of each protein identified is provided in supplemental Table S1.

### Statistical Analyses of DEPs

Statistical analyses were performed using IBM SPSS, version 25.0, for quantitative comparison of proteins in EVs derived from AML and control cells. According to the normality result by the Shapiro–Wilk test, the parametric continuous data were statistically analyzed using one-way ANOVA test with a *p*-value, followed by the Bonferroni multiple comparison post hoc test. To intuitively understand the function of the differentially expressed genes in each AML cell line, the relationship of differentially expressed genes in the AML cell line was visualized. Compared with two types of control cells, DEPs from EVs of each AML cell line were screened depending on a threshold *p* < 0.05 and |logFC| > 0.58, corresponding to a fold change (FC) > 1.5 or <0.67. DEPs commonly selected from EVs derived from each of the three AML cell lines were analyzed for Gene Ontology Biological Process and Kyoto Encyclopedia of Genes and Genomes pathways using DAVID software (32).

### Network Construction Between Kinases and Functional Node Activity

A kinase–kinase interaction network was constructed by analyzing the biological function ontologies of kinase expressed in AML and control cells. Based on the biological process analyzed of kinases, STRING software was used to predict the possible interaction network between proteins (33). Expected protein–protein interaction networks with a score of 0.4 for all kinases were plotted using Cytoscape software (34). Interaction networks had distinct biological processes between constituent genes, and each node was represented by color change and size, respectively, based on FC and *p*-value between AML and control cells.

### ELISA

To identify changes in CD markers before and after treatment, EVs isolated from the BM of patients with AML were evaluated using a Human CD47 ELISA Kit (MyBioSource) and an ExoTEST CD53-exosome ELISA Kit (HansaBioMed, Tallinn, Estonia). ELISAs were performed according to the manufacturer's instructions.

### Cell Viability Assay

The following drugs were used: nilotinib (Sigma-Aldrich; catalog no. CDS023093), acalabrutinib (AdooQ Bioscience; catalog no. A15824), and fostamatinib (APExBio; catalog no. B2284). EZ-Cytox (DoGen) was used for all cell viability assays that were performed according to the manufacturer's instructions. Absorbance at 450 nm was measured using a SpectraMax Plus 384 plate reader (Molecular Devices Corporation). All experiments were performed in triplicate and repeated three times.

### Statistical Analysis of Correlation of Survival Rates and AML-Derived EV Markers

The median values and ranges or means and SDs for continuous variables and percentages for categorical values were used. First, we analyzed the correlation between survival and mRNA expression data of initial diagnosis for CD53 and CD47 from TCGA database (https://portal.gdc.cancer.gov/) (35). We set a cut-off value of fragments per kilobase of transcript per million (FPKM) of CD53 and CD47 with the highest hazard ratio (HR) and the lowest *p*-value to analyze the survival probability according to these markers. The survival rate of the divided group with these cut-off values was analyzed with the Kaplan–Meier analysis using the log-rank test. The HR was estimated using multivariable Cox regression (36). Second, we analyzed the correlation between survival and the expression levels of CD53 and CD47 measured by ELISA in EVs from the BM of patients with AML before and after remission induction treatment. Relapse-free survival (RFS) was defined as the time from diagnosis to relapse or death and was calculated according to the Kaplan–Meier method using the log-rank test. We used the R program, version 3.6.1, and IBM SPSS, version 25.0, software to analyze the data. A *p*-value < 0.05 was considered to indicate a significant difference.

## Results

### Validation of EVs Derived From AML and Control Cells

**Fig. 1:**
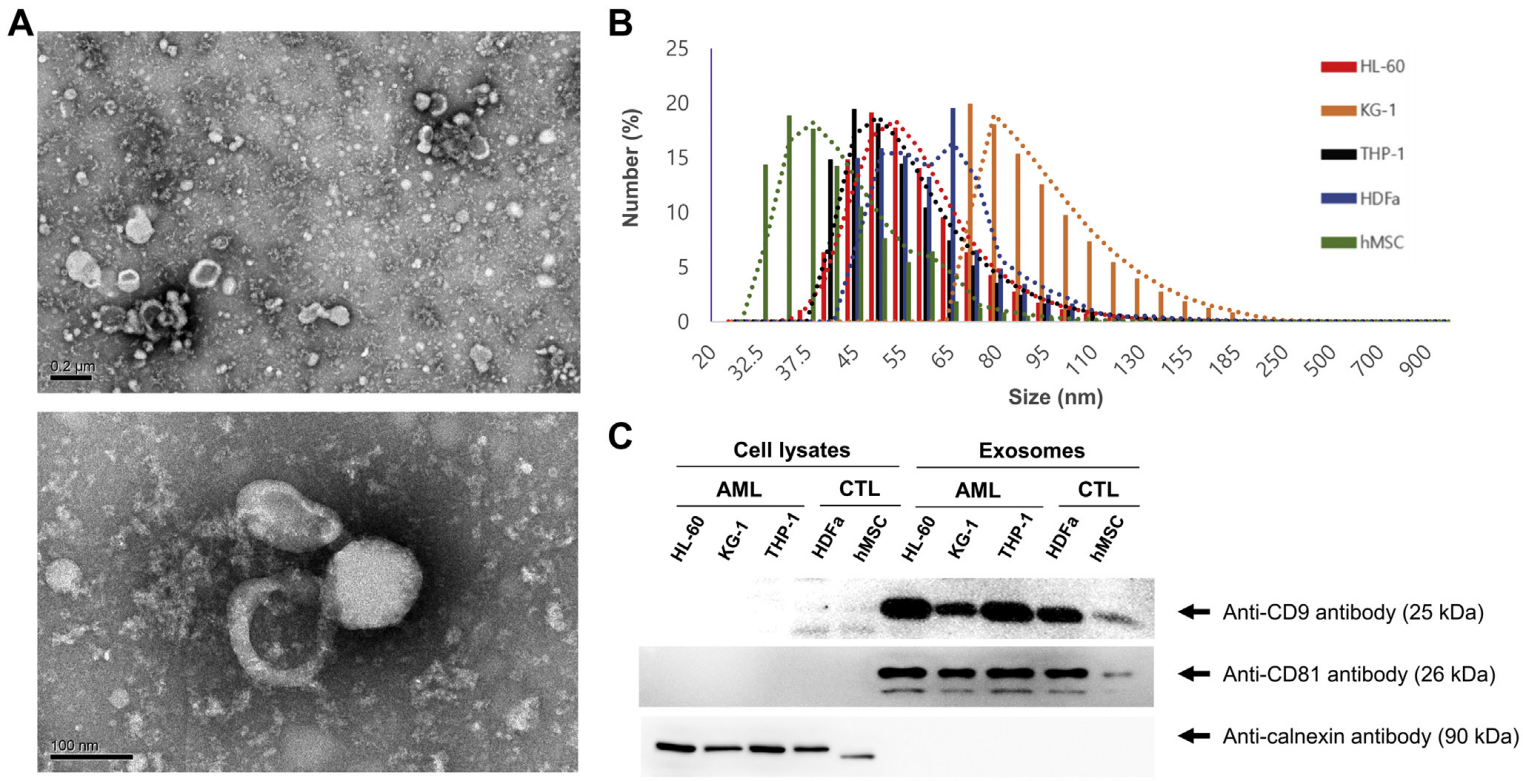
**Validation of extracellular vesicles (EVs) derived from acute myeloid leukemia (AML) cells and control cells**. *A*, in transmission electron microscopy, the size of the cell line–derived EVs was <200 nm. They were visualized as cup-shaped vesicles. *B*, dynamic light scattering revealed a size range of 30 nm to 185 nm. *C*, a total of 30 μg of each protein sample was separated using 10% SDS-PAGE. Western blot analysis showed that the isolated EVs were positive for the exosome markers (CD9 and CD81) but negative for the endoplasmic reticulum marker calnexin.

The properties of EVs isolated by size-exclusive chromatography from supernatants of AML or control cell lines are shown in Figure 1. TEM revealed that the size of EVs from AML and control cells was <200 nm. They were visualized as cup-shaped vesicles under high magnification (Fig. 1*A*). The size distribution of these EVs measured by DLS indicated a range between 30 nm and 185 nm (Fig. 1*B*). Western blotting showed that the isolated EVs were positive for the exosome markers (CD9 and CD81) and negative for the endoplasmic reticulum marker calnexin (Fig. 1*C*).

### Protein Identification and Quantification

**Fig. 2:**
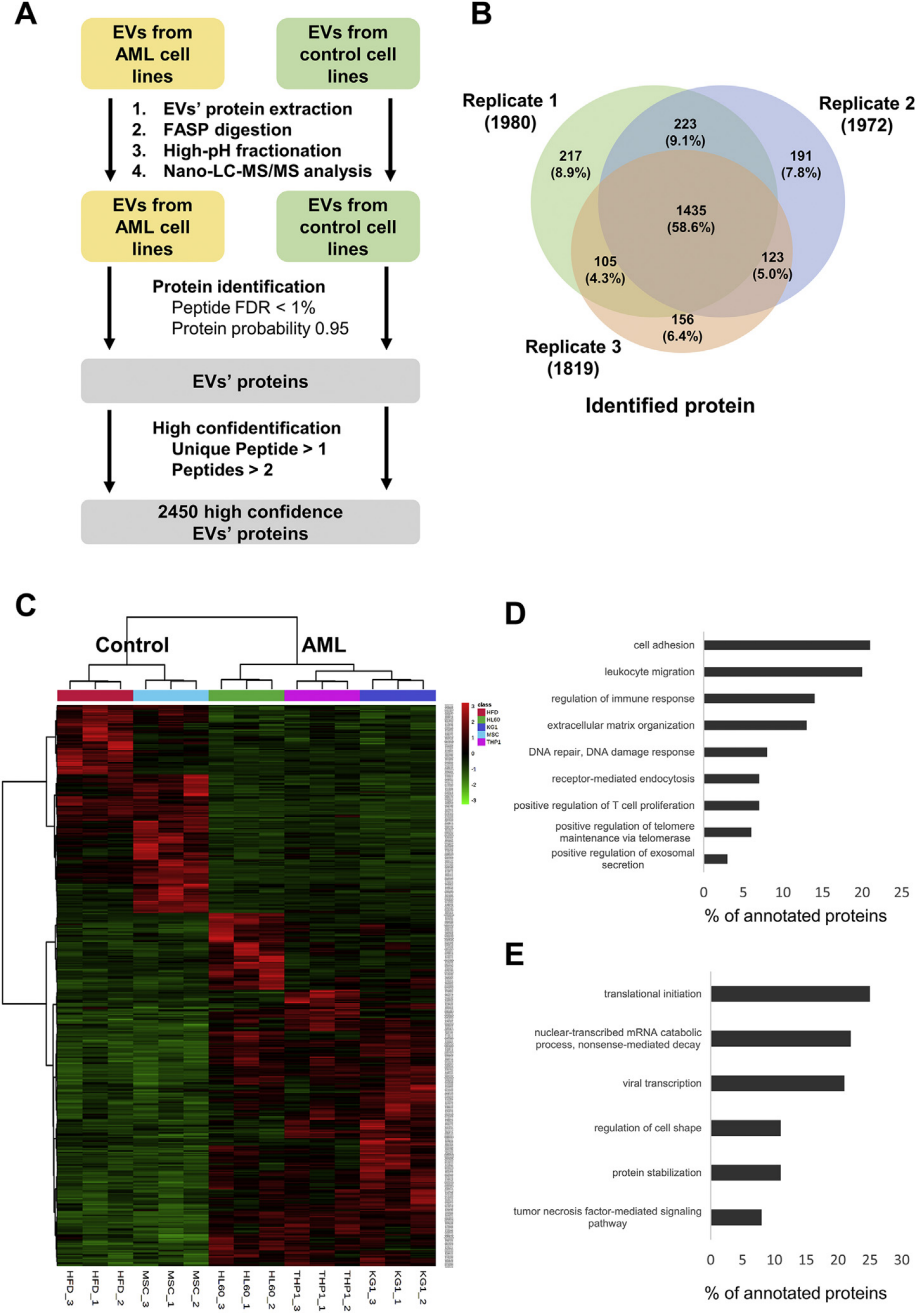
**Proteins identified by LC-MS/MS analysis and protein quantification.***A*, quantitative proteomic analysis of the EVs isolated from AML cells (HL-60, KG-1, and THP-1) and control cells (HDFa and hMSCs). *B*, the Venn diagram showing the overlapping proteins from the protein profiles of three replicates. A scheme of the proteomic approach for the identification of EV proteins. *C*, hierarchical clustering of differentially expressed proteins (DEPs) among EVs derived from AML cell lines. The heat map graphically represents the proteins from the EVs derived from AML cell lines whose expression levels were significantly different compared with those derived from control cell lines. *D* and *E*, functional analysis of DEPs was conducted using DAVID software to analyze gene ontology (GO) to identify biological processes. Upregulated biological processes in EVs derived from AML cell lines compared with EVs derived from control cell lines (*D*) and downregulated biological processes in EVs derived from AML cell lines versus EVs derived from control cell lines (*E*). Note that proteins generally have several GO annotations (annotated proteins *p*-value < 0.05). AML, acute myeloid leukemia; EVs, extracellular vesicles; HDFa, human dermal fibroblasts, adult; hMSCs, human mesenchymal stem cells; LC-MS/MS, liquid chromatography–tandem mass spectrometry.

To analyze the EVs from HL-60, KG-1, and THP-1 AML cell lines and control cells (HDFa and hMSCs), quantitative and comparative proteomic analyses were performed using LC-MS/MS. To determine the proteomic profiles of the EVs from AML cells, LC-MS/MS was performed after filter-aided sample preparation digestion and subsequent fractionation. Proteins from the isolated EVs were digested and fractionated to form peptides for MS examination. To improve protein confidence, the analyzed protein levels were tested against the 1% FDR threshold and identified with at least one unique peptide (Fig. 2*A*). After triplicate runs, the acquired independent MS/MS spectra were searched using the SEQUEST HT algorithm in Proteome Discoverer 2.4 against the Human UniProtKB database.

The overlapping proteins in all three replicate proteomic profiles were plotted in a Venn diagram. In total, 1980, 1972, and 1819 proteins from subjects 1, 2, and 3, respectively, were identified. A total of 2450 unique proteins were identified by LC-MS/MS analysis from all three independent replicates (Fig. 2*B*).

Based on the EV data from the five cell types, the significance level was determined by performing ANOVA and post hoc Bonferroni tests to select DEPs. All overlapping DEPs between the control and AML groups were selected with a cut-off value established at an FC of more than 1.5 and less than 0.67 and significance at 95% confidence interval (CI). The heat map was represented by the expression levels of the DEPs (Fig. 2*C*). In total, 461 proteins were differentially expressed in AML cell–derived EVs. Of the 461 DEPs, 290 were upregulated and 171 were downregulated. Hierarchical clustering of the DEPs was performed as shown in Figure 2*C*. Functional analysis of DEPs was conducted using DAVID software, which performs gene ontology analyses for biological processes. Analysis of the biological processes associated with the upregulated proteins revealed an enrichment of the pathways associated with extracellular matrix organization, cell adhesion, and positive regulation of exosomal secretion (Fig. 2*D*). The selected DEPs were also confirmed to be related to the major pathways of leukocyte migration, regulation of immune response, DNA repair, DNA damage response receptor-mediated endocytosis, positive regulation of T-cell proliferation, and positive regulation of telomere maintenance via telomerase. Pathways associated with translational initiation, nuclear-transcribed mRNA catabolic process, nonsense-mediated decay, viral transcription, regulation of cell shape, protein stabilization, and tumor necrosis factor–mediated signaling pathways were represented in the downregulated proteins (Fig. 2*E*). The highly expressed proteins identified were interrelated, which was supported by the significantly high protein–protein interactions characteristic of AML biological processes. Proteins generally had several gene ontology annotations (annotated proteins *p* < 0.01).

The biological processes of the DEPs in AML and control cell line–derived EVs are listed in Table 2. Among the upregulated proteins, proteins were selected if expression has been reported in AML cells, hematopoietic stem cells, or leukocytes, except those already used clinically, and the proteins were surface-exposed and capable of detection by common clinically used methods, such as ELISA and flow cytometry. The CD4, CD53, CD33, and CD47 surface proteins were selected as potential biomarker candidates for further analysis (37, 38, 39, 40, 41).

**Table 2:** Gene ontology analysis of proteins in AML cell line–derived EVs

Protein	Accession number	Gene symbol	AML cell–derived EV group	Fold change			ANOVA *p*-value
				Normalized with HDFa	Normalized with hMSCs	AML/control	
Upregulated proteins in AML cell line–derived EVs (*p*-value < 0.05, FC > 1.5)							
T cell surface glycoprotein CD4	P01730	CD4	KG1	19.149∗	5.161∗	10.221	<0.01
			HL60	17.993	7.007		
			THP1	5.493	6.584		
Leukocyte surface antigen CD53	P19397	CD53	KG1	8.976	6.738	7.664	0.032
			HL60	9.483	7.024		
			THP1	6.378∗	7.42∗		
Leukocyte antigen CD37	P11049	CD37	KG1	1.868	15.208	7.175	0.031
			HL60	2.002∗	4.745∗		
			THP1	14.193	5.084		
Lumican	P51884	LUM	KG1	4.568∗	4.801∗	4.628	<0.01
			HL60	5.108∗	4.288∗		
			THP1	4.293∗	4.795∗		
Myeloid cell surface antigen CD33	P20138	CD33	KG1	3.390	3.145∗	3.218	0.043
			HL60	4.946	2.590		
			THP1	2.155	3.779		
Integrin beta-7	P26010	ITGB7	KG1	4.449∗	1.622∗	2.931	<0.01
			HL60	3.897	3.114		
			THP1	1.852	2.727		
Leukosialin	P16150	SPN	KG1	2.844	3.018	2.717	0.017
			HL60	2.757∗	2.321		
			THP1	3.113	2.250		
Leukocyte surface antigen CD47	Q08722	CD47	KG1	1.017∗	3.053∗	1.974	<0.01
			HL60	1.331∗	1.873∗		
			THP1	2.332	2.452		
Nucleophosmin	P06748	NPM1	KG1	4.244∗	5.964∗	4.041	<0.01
			HL60	3.749∗	5.268∗		
			THP1	2.383	3.348		
Downregulated proteins in AML cell line–derived EVs (*p*-value < 0.05, FC <0.67)							
Reversion-inducing cysteine-rich protein with Kazal motifs	O95980	RECK	KG1	0.336∗	0.975	0.595	<0.01
			HL60	0.617	0.511		
			THP1	0.531	0.938		
Peroxidasin homolog	Q92626	PXDN	KG1	0.530	0.407∗	0.490	0.019
			HL60	0.345	0.707		
			THP1	0.626	0.460		
Nidogen-2	Q14112	NID2	KG1	0.298∗	0.759	0.421	<0.01
			HL60	0.905∗	0.292		
			THP1	0.250∗	0.888		
Sushi repeat-containing protein SRPX	P78539	SRPX	KG1	0.484∗	0.318∗	0.394	<0.01
			HL60	0.299∗	0.550∗		
			THP1	0.515∗	0.339∗		
Lysosome membrane protein 2	Q14108	SCAR-B2	KG1	0.381	0.319	0.380	0.043
			HL60	0.402∗	0.427		
			THP1	0.303	0.450		
Pappalysin-1	Q13219	PAPPA	KG1	0.278	0.298∗	0.348	<0.01
			HL60	0.210	0.541∗		
			THP1	0.394	0.41∗		
Myoferlin	Q9NZM1	MYOF	KG1	0.276∗	0.300∗	0.298	<0.01
			HL60	0.262∗	0.326∗		
			THP1	0.317∗	0.309∗		

AML, acute myeloid leukemia; EV, extracellular vesicle; FC, fold change; HDFa, human dermal fibroblasts, adult; hMSCs, human mesenchymal stem cells. The post-hoc test was performed through Bonferroni multiple comparison, and the *p*-value for that was displayed through asterisks on the bar graph (∗*p* ≤ 0.05).

### Evaluation of the Selected Protein Biomarkers

**Fig. 3:**
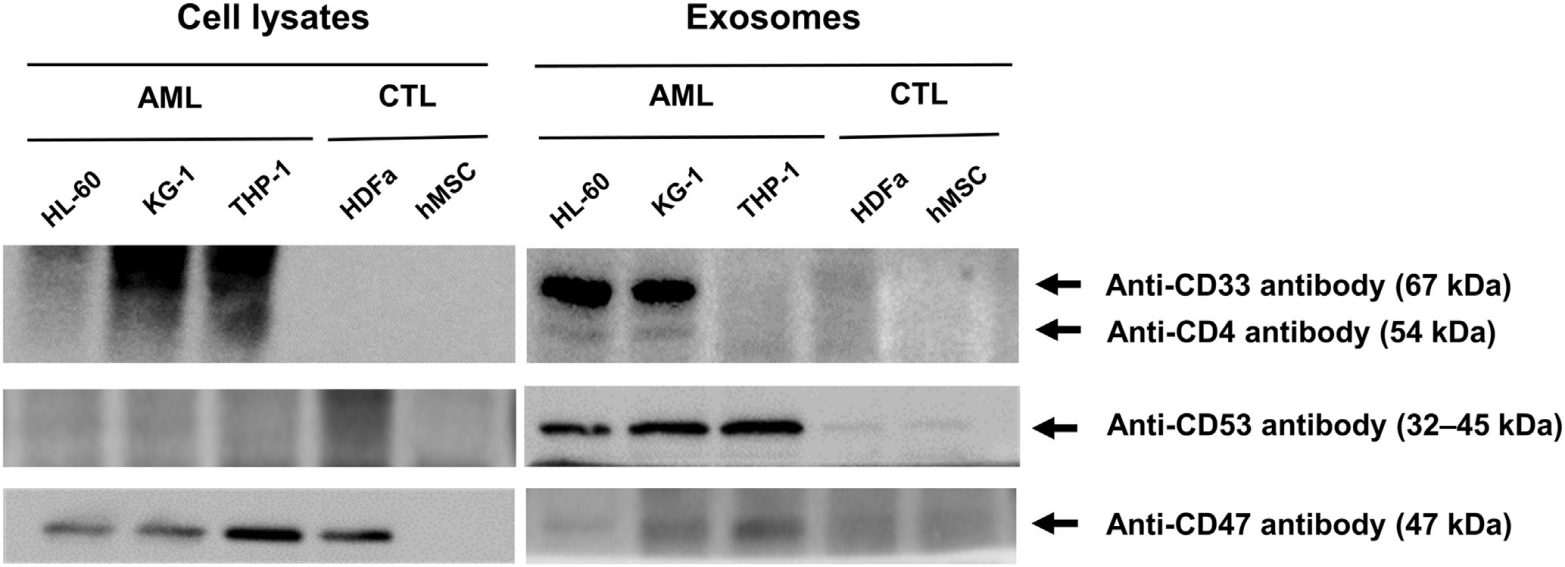
**Validation of selected EV protein biomarkers**. Among the top-ranked upregulated DEPs, surface antigens CD33, CD4, CD53, and CD47 were selected as AML protein biomarker candidates for further analysis. CD33, CD4, CD53, and CD47 were expressed in EVs from AML cell lines but not in EVs from control cell lines. CD53 and CD47, upregulated in all three AML cell lines, were selected as the final candidates for the use as biomarkers. AML, acute myeloid leukemia; EVs, extracellular vesicles; DEPs, differentially expressed proteins.

The differential expression of CD4, CD53, CD33, and CD47 was verified by Western blotting (Fig. 3). CD4, CD33, CD53, and CD47 were expressed in EVs from AML cell lines but not in EVs from the control cell lines. CD53 and CD47, which were expressed in all three AML cell lines, were selected as the final candidates to investigate whether these enriched proteins could be potential biomarkers in patients with AML. Two assessment approaches were used.

**Fig. 4:**
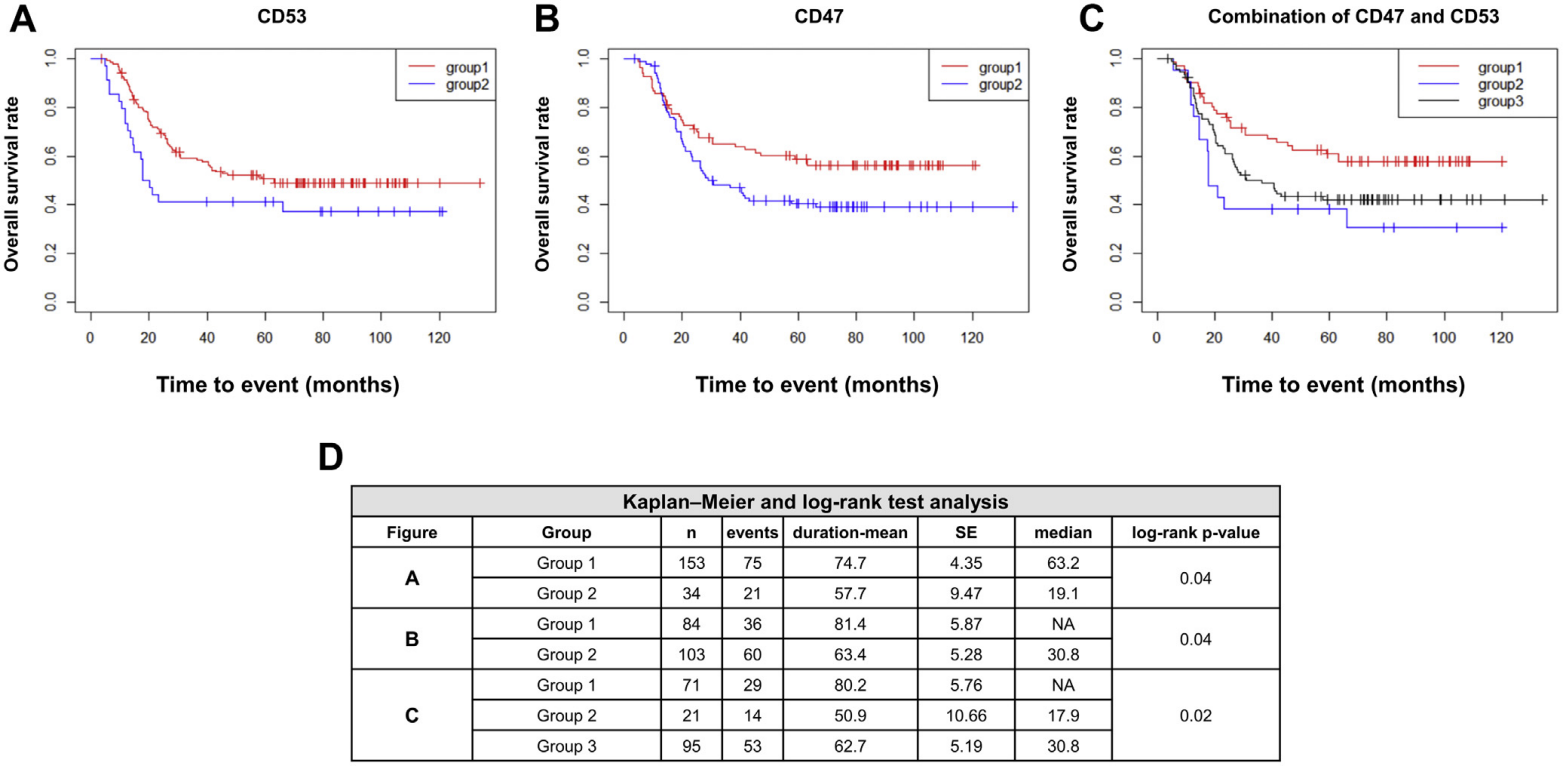
**Survival analysis of the selected biomarkers from patients with AML based on The Cancer Genome Atlas data**. *A*, Kaplan–Meier survival curves showed the correlation between the survival and mRNA expression data of CD53 and CD47 at initial diagnosis based on TCGA data of 187 patients with AML. The patients were divided into two groups according to an effective cut-off value. If the CD53 or CD47 expression was lower than the cut-off value, patients were classified into group 1. If not, they were classified into group 2. In case of CD53, group 2 showed decreased survival probability with borderline significance relative to group 1. *B*, in the case of CD47, the survival probability of group 2 was significantly lower than that of group 1. *C* next, we analyzed the survival probability by combining two markers and reclassifying patients into three groups. Group 1 was composed of patients with AML referred to group 1 with regard to both markers. Group 2 was composed of patients with AML referred to group 2 with regard to both markers. Patients with AML not belonging to group 1 or 2 were classified into group 3. Group 2 showed the lowest survival probability relative to group 1 or group 3. *D*, Kaplan–Meier survival analysis with log-rank test for overall survival of patients with AML according to CD53 and CD47 is shown in Table 3. AML, acute myeloid leukemia; TCGA, The Cancer Genome Atlas.

First, we investigated the survival of patients with AML using information from TCGA database. The survival and mRNA expression data of CD53 and CD47 at initial diagnosis were obtained and a total of 187 patients with AML were analyzed. In the case of CD53, the effective cut-off value of FPKM was 120 (minimum *p* = 0.015). We divided the patients into two groups according to the FPKM value. If CD53 expression was <120, the patients were classified into group 1. If not, the patients were classified into group 2. Group 2 showed a decreased survival probability with borderline significance when compared with group 1 (group 1: *n* = 153, group 2: *n* = 34, HR: 1.62, 95% CI: 0.99 to 2.63, *p* = 0.051) (Fig. 4*A* and Table 3). In the case of CD47, the effective cut-off value of FPKM was 13 (minimum *p* = 0.018), and patients were divided into two groups, as was the case with CD53. The survival probability of group 2 was significantly lower than that of group 1 (group 1: *n* = 84, group 2: *n* = 103, HR: 1.54, CI: 1.01–2.34, *p* = 0.041) (Fig. 4*B* and Table 3). Next, we analyzed the survival probability by combining two markers and reclassifying the patients into three groups: Group 1 was composed of patients with AML referred to group 1 in case of both markers. Group 2 was composed of patients with AML referred to group 2 in case of both markers. Patients with AML who could not be classified into groups 1 and 2 were classified into group 3. Group 2 showed the lowest survival probability relative to group 1 (group 1: *n* = 71, group 2: *n* = 21, HR: 2.43, CI: 1.28–4.63, *p* = 0.006) and group 3 (group 2: *n* = 21, group 3: *n* = 95, HR: 1.64, CI: 1.03–2.57, *p* = 0.033) (Fig. 4*C* and Table 3).

**Table 3:** Multivariable Cox regression analysis of individual or combined model for CD53 and CD47 levels

Model	Variable	HR	95% CI	*p*-value
Individual model	Age	1.04	1.00–1.07	0.0232
	Female (reference male)	1.45	0.96–2.18	0.0743
	**CD53 > 120 (ref. CD53 ≤ 120)**	**1.62**	**0.99**–**2.63**	**0.0513**
	**CD47 > 13 (ref. CD47 ≤ 13)**	**1.54**	**1.01**–**2.34**	**0.0411**
Combined model	Age	1.04	1.00–1.07	0.0232
	Female (reference male)	1.44	0.96–2.17	0.0759
	**CD53 > 120 and CD47 > 13 (ref. CD53 ≤ 120 and CD47 ≤ 13)**	**2.43**	**1.28**–**4.63**	**0.0064**
	**CD53 ≤ 120 or CD47 ≤ 13 (ref. CD53 ≤ 120 and CD47 ≤ 13)**	**1.63**	**1.03**–**2.57**	**0.0333**

Bold indicates CD markers to be verified in this study. CI, confidence interval; HR, hazard ratio.

**Fig. 5:**
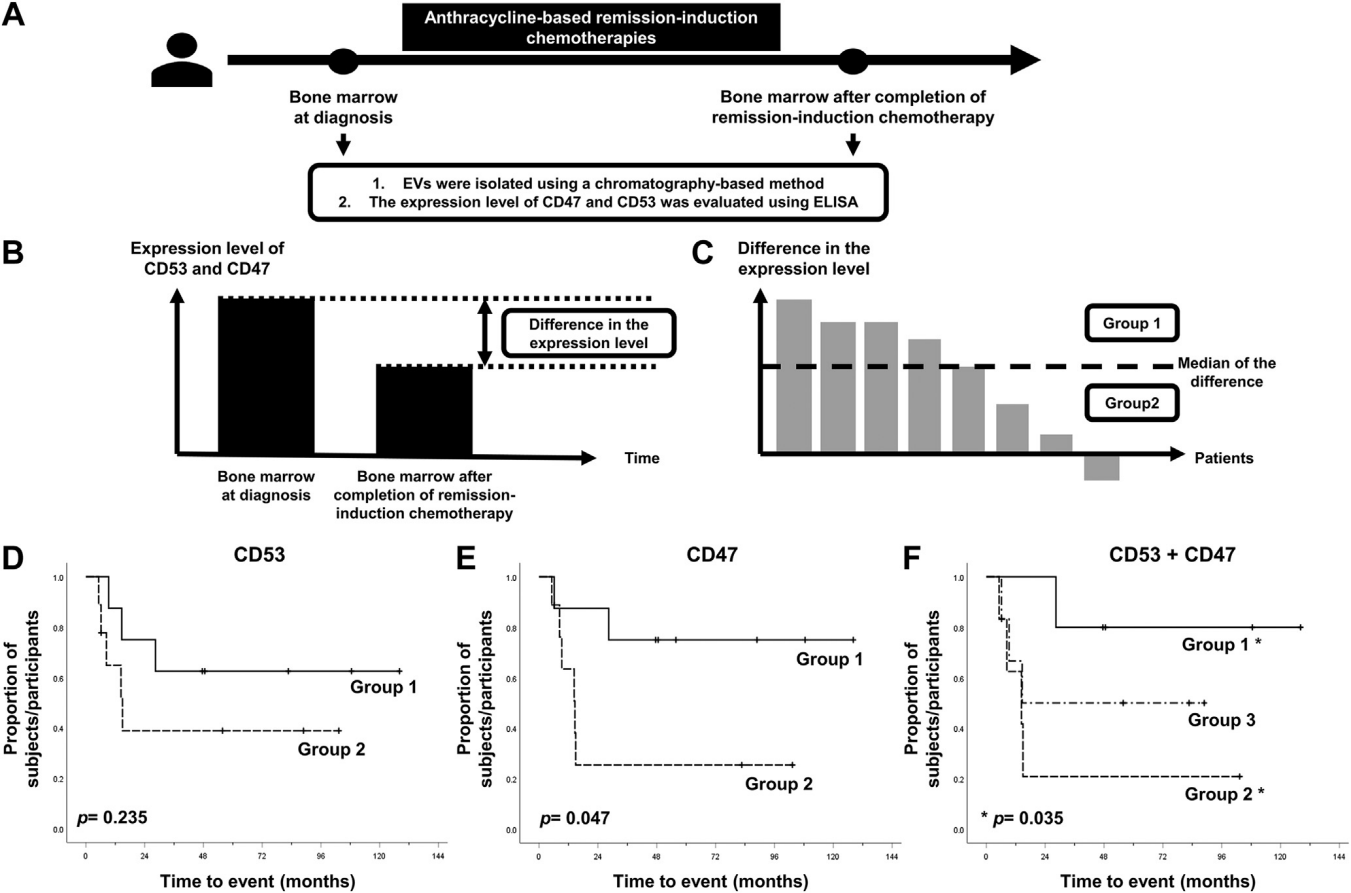
**Evaluation of selected protein biomarkers from EVs derived from bone marrow of patients with AML**. *A–C*, the expression levels of selected markers (CD53 and CD47) in bone marrow (BM) EVs of patients with AML at the time of diagnosis and after treatment were measured using ELISA. All patients achieved CR after anthracycline-based remission induction chemotherapy. The changes in the expression levels of CD53 and CD47 between diagnosis and after treatment were determined. If the difference in the expression levels of CD53 or CD47 between two time points was greater than the median, the patients were classified into group 1. If not, they were put into group 2. *D*, the levels of CD53 decreased after treatment, compared with the levels during diagnosis; relapse-free survival (RFS) tended to be longer, but there was no significant difference (group 1; n = 8 *versus* group 2; n = 9, *p* = 0.235). *E*, in the case of CD47, RFS was significantly longer in group 1 in which CD47 was significantly decreased after treatment (group 1; n = 8 *versus* group 2; n = 9, *p* = 0.047). *F*, when these two markers were combined for analysis, the prognosis could be further subdivided. According to changes in the expression levels of CD53 and CD47, patients were classified into three groups. If the patients showed decreased values compared with the median CD53 and CD47 levels, they were classified into group 1 (n = 5), and if the patients did not show a decrease in CD53 and CD47 levels, they were classified into group 2 (n = 6). Patients who could not be categorized into either group 1 or group 2 were classified into group 3 (n = 6). RFS was better predicted when CD53 and CD47 were used together than when CD53 or CD47 was used alone (group 1; n = 5 *versus* group 2; n = 6, *p* = 0.035). AML, acute myeloid leukemia; CR, complete remission; EVs, extracellular vesicles.

Second, the expression levels of selected markers (CD53 and CD47) in EVs from the BM of patients with AML at the time of diagnosis and after treatment were measured by ELISA. We analyzed the correlation between the differences in expression levels of the two markers (before and after chemotherapy) and RFS to understand the prognosis after treatment. All patients achieved CR after anthracycline-based remission induction chemotherapy. The implications of changes in the expression levels of CD53 or CD47 between the time of diagnosis and after treatment were determined. If the difference in expression levels of CD53 or CD47 between the two time points was greater than the median, the patient was classified into group 1. If not, they were put into group 2 (Fig. 5, *A*–*C*). If the expression levels of CD53 decreased after treatment, RFS tended to be longer, but there was no significant difference (group 1; *n* = 8 *versus* group 2; *n* = 9, *p* = 0.235) (Fig. 5*D*). In the case of CD47, RFS was significantly longer in the group in which CD47 was significantly decreased after treatment (group 1; *n* = 8 *versus* group 2; *n* = 9, *p* = 0.047) (Fig. 5*E*). When these two markers were combined, we were able to further subdivide the categories for prognosis. According to changes in the expression levels of CD53 and CD47, patients were divided into three groups. If patients had decreased values for both CD53 and CD47, they were categorized as group 1 (*n* = 5). If patients did not show any decrease in either CD53 or CD47, they were categorized as group 2 (*n* = 6). Patients who could not be categorized as either group 1 or 2 were categorized as group 3 (*n* = 6). RFS was significantly better when both CD53 and CD47 were used together than when either marker was used alone (group 1; *n* = 5 *versus* group 2; *n* = 6, *p* = 0.035) (Fig. 5*F*).

### Evaluation of Identified Kinases and Their Interactions

**Fig. 6:**
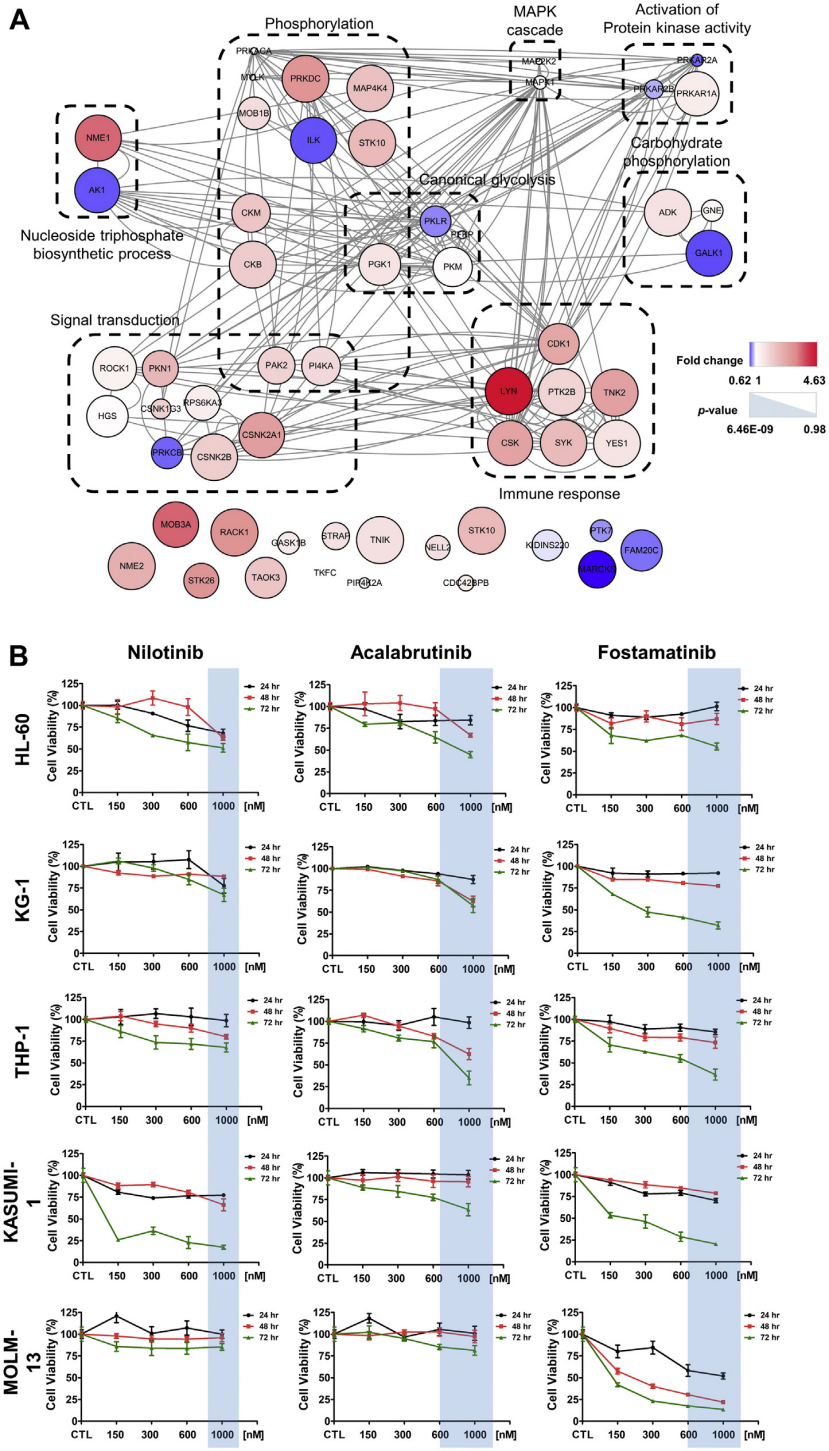
**Network modeling of kinases in AML cell line–derived exosomes**. *A*, network model analysis showed the activity of various kinases regulated in EVs derived from AML cell lines. These were enriched in various critical biological cellular signaling pathways, including cellular processes involving immune response, signal transduction, activation of protein kinase activity, canonical glycolysis, carbohydrate phosphorylation, phosphorylation, nucleoside triphosphate biosynthetic process, and MAPK cascade. Each node is represented by color change and size, based on fold change and *p*-value, respectively, between AML and control cells. Node colors represent proteins that were more greatly decreased (*blue*) or increased (*red*) in AML cell line–derived EVs than in control cell–derived EVs. The larger the node size, the smaller the *p*-value, indicating significance. The connected lines (*gray edges*) between the nodes represent the physical interactions or regulation between proteins in the network. *B*, the clinically applicable chemotherapy drugs, nilotinib, acalabrutinib, and fostamatinib, which block LYN, CSNK2A1, and SYK proteins, that showed the highest increase in expression in our study were used to treat AML cell lines for 24 h, 48 h, or 72 h. All three drugs inhibited proliferation of the AML cell lines (HL-60, KG-1, THP-1, Kasumi-1, and MOLM-13) at maximum serum concentrations below those used in the clinical treatment (*blue box*). Fostamatinib showed the greatest inhibitory effect among the drugs. AML, acute myeloid leukemia; EVs, extracellular vesicles; MAPK, mitogen-activated protein kinase.

Kinases maintain various cellular functions and interact closely with each other to establish a network to regulate biological activity. Compared with the control group, kinases with increased expression in AML regulated cell signaling in cancer related to the origin and dissemination disease-specific pathology. A total of 56 kinase proteins were identified in the AML and control cell types. Of these, 39 had close interactions with each other (Fig. 6*A*), with important roles in a variety of important biological signaling pathways, including immune responses, signaling, activation of protein kinase activity, glycolysis, and cellular processes associated with the mitogen-activated protein kinase cascade (Fig. 6*A*). The FC of AML cell–derived EV normalized to the control group was calculated, and the *p*-value was obtained by performing a one-way ANOVA, followed by the Bonferroni multiple comparison test (supplemental Table S2). Lyn, SYK, CSK, CSNK2A1, and PTK2B were identified as kinase proteins whose FC was >1.5 times (*p* < 0.05). These kinases are involved in the immune response and signal transduction pathways.

Among the clinically available drugs, nilotinib, acalabrutinib, and fostamatinib block Lyn, CSNK2A1, and SYK proteins, which were the most represented in our analysis of AML cell–derived EVs. These drugs were used to treat AML cell lines for 24, 48, or 72 h. We also compiled literature data on blood concentration levels for nilotinib, acalabrutinib, and fostamatinib in humans so that we could evaluate the clinical suitability. When used in a clinical setting, the maximum serum concentration was in the range of 1000 to 4000 nM for nilotinib (42), 600 to 2000 nM for acalabrutinib (43, 44), and 668 to 1020 nM for fostamatinib (45). All three drugs had an inhibitory effect on the proliferation of the HL-60, KG-1, THP-1, Kasumi-1, and MOLM-13 AML cells at concentrations lower than the reported maximum serum concentrations (Fig. 6*B*). Inhibition of proliferation was the strongest for fostamatinib.

## Discussion

We isolated and highly purified AML cell–derived EVs and identified specifically enriched proteins using MS. CD53 and CD47 were selected from the upregulated proteins in AML cell–derived EVs. Their enhanced expression at diagnosis of patients with AML was associated with reduced survival. Their reduced expression after treatment might be related to RFS. Kinase interaction networks provide a systematic understanding of the biological context, function, and regulation in cells. In particular, kinases with greater FCs in AML cell–derived EVs than in control cells are involved in the signaling network related to cancer and are often a driving force of disease. Therefore, treatment with inhibitors that target these kinases can block many factors related to cancer progression. We also selected drugs (nilotinib, acalabrutinib, and fostamatinib) that block LYN, CSNK2A1, and SYK proteins, which were the most enriched in the EVs derived from the AML cells, to evaluate their potential clinical applications. All three drugs reduced the viability of the AML cells at clinically relevant concentrations.

Proteomics is a protein analysis tool that can be used to interpret how genes function in their environment. Therefore, proteomics could become a powerful tool to evaluate and identify biomarkers based on the analysis of dynamic protein changes in patients with cancer exposed to various treatment environments, including surgery, chemotherapy, and radiation therapy (46). However, cell-based proteomics has limitations in clinical applications because of the huge amount of data generated as a result of the heterogeneity of cancer cells and the dynamic changes in their environment (47). EV-based proteomics could be one of the solutions to these limitations. The total amount of EVs is higher in patients with cancer than in healthy controls (48). In addition, EV sorting based on proteins, RNAs, and DNAs from the original cells can be performed (49). As EVs can be obtained from any bodily fluid and the total amount of information they possess is less than that of their originating cells, they can be tested in a clinically relevant manner (50). In previous studies, proteomic analysis has been used to suggest biomarker candidates from cancer-specific EVs generated from a variety of solid tumors, including breast, colon, and lung cancers (51). In this study, we established that a similar approach using AML cell–derived EVs can be applied to novel drug and biomarker identification.

Although the precise function of EVs remains unknown, they reflect the phenotypic state of the cells that generate them and contain all the known molecular constituents of these cells, including proteins, RNAs, and DNAs (52). Thus, EVs are a potential source of information to identify new biomarkers and therapeutic targets for various cancers. Previous studies reported that glypican-1–positive circulating exosomes might be diagnostic and prognostic biomarkers for the early detection of pancreatic cancer (16). Another study reported that exosomes from drug-resistant breast cancer cells contain miRNAs that could transmit chemoresistance, making them a potential therapeutic target, which may enhance a patient's response to therapy (17). Thus, there have been few studies focusing on the roles of AML-derived EVs and their potential applications in clinical diagnostics, prognosis, and therapy. In this study, we demonstrated that the EVs derived from AML cells could be a useful platform to develop biomarkers and identify novel drug targets in AML.

We confirmed the positive AML cell–derived EV biomarker by referring to previous studies on EVs including microvesicles and exosomes derived from AML. The positive AML cell–derived EV biomarkers identified in our experimental data were CD13, CD33, CD34, NPM1, and TGFβ1, as previously reported (18, 20, 53, 54, 55). Presently, CD33 and NPM1 were significantly increased in all AML groups compared with the control group (*p* < 0.05). However, compared with the control group, CD34 expression was increased only in EVs derived from THP1 cells. The expression was decreased in other AML cell–derived EVs, and CD13 and TGFβ1 were decreased in all AML cell groups compared with control cells. These discrepancies may result from different experimental conditions, including EV preparation, analytical equipment, and statistical methods. The AML cell–derived EV markers selected in our study included positive markers that were previously identified in AML cells or hematopoietic stem cells in previous studies (18, 20, 37, 38, 39, 40, 41, 53, 54, 55).

Among the DEPs in AML cell–derived EVs, 62 proteins were previously identified in whole-cell studies (56, 57, 58, 59). This discrepancy strongly suggests the benefit of EV research, as EVs reflect the dynamic changes of cell states. Interestingly, the EV proteins proposed as candidate biomarkers, including CD47, CD33, CSK, LYN, and SYK, agreed well with results from whole cells. However, some candidate biomarkers, such as CSNK2A1, have shown opposite protein-expression patterns in cells and EVs (56).

Among the selected markers, CD53 and CD47 were significantly upregulated in AML cell–derived EVs and were associated with the survival of patients with AML. TCGA database is a comprehensive atlas of cancer genomic profiles and provides data for genome, transcriptome, and proteome including clinical metadata (60). We used TCGA database to validate the levels of CD53 and CD47, which were selected as biomarkers in this study. Transcriptome data analysis revealed that higher levels of CD53 and CD47 at diagnosis were associated with lower survival rates. In addition, we further measured the levels of CD53 and CD47 at diagnosis and after treatment using ELISA. The analysis revealed a lower risk of recurrence if values of CD53 and CD47 in EVs were lower after treatment. Collectively, the results of this study suggest that proteomic approaches using AML cell–derived EVs could be useful platforms for biomarker research to predict patient survival and measure minimal residual disease.

One of the most interesting observations in this study is that drugs selected based on protein expression in AML cell–derived EVs reduced the viability of parental AML cells. The network model analysis of DEPs demonstrated a prominent increase in Lyn, CSNK2A1, SYK, CSK, and PTK2B kinase protein expression. Lyn interacts with and phosphorylates tyrosine residues in SYK and BTK kinases (61). Based on this information, tyrosine kinase inhibitors targeting these kinases (nilotinib, acalabrutinib, and fostamatinib) were selected for evaluation. In general, nilotinib is used for chronic myeloid leukemia or Bcr-Abl–expressing hematological malignancies, acalabrutinib is used for chronic lymphocytic leukemia or B cell lymphoma, and fostamatinib is used in the treatment of autoimmune diseases. The effect of these drugs in the treatment of AML is unclear. However, several studies have suggested the potential of nilotinib as a therapeutic agent for AML that features BCR-ABL1 transcription (62, 63, 64). The potential of fostamatinib as a therapeutic agent in FLT3-ITD–positive AML has been supported by evidence of the importance of SYK in the regulation of FLT3 (59). However, these studies were conducted using leukemia cell–based gene analysis, which selected the drugs by simple genotyping and not by functional analysis of that gene. In this study, drugs that could affect the original AML cell lines were selected by analyzing the proteome of EVs. In our opinion, the success of these drugs against parental cell lines suggests that EVs might be key to understanding the crucial oncological features of AML.

There are several limitations to this study. First, there are still various challenges to analyze biomarkers with EVs (65, 66). The absence of a standardized procedure for EV isolation is one challenge. Because the EV isolation method affects the physical and molecular properties of the isolated EV, standardization is needed to enable comparison between studies and to improve the reproducibility of results. Collection and utilization at the clinical stage is another challenge. Because various treatments have been applied to patients, it is very difficult to determine biomarkers using EVs collected from patients. In addition, the characteristics of EVs collected from patients are also affected by the treatment applied to the patient. To avoid these challenges, we first compared EVs from cell lines. The second limitation is that EVs derived from AML cell line– and control cell line–derived EV proteins were identified and quantified by tagging each sample with TMT. Protein identification was then performed using the reporter-ion quantification method based on the TMT for each sample. TMT labeling may produce lesser protein profiling information than label-free quantification methods (67). The label-free approach has been reported to have a higher number of confidently identified proteins than the TMT approach and generates more information for protein profiling using at least two peptides for identification. In addition, the protein profile information may vary between samples in the label-free approach. The label-free approach could be useful to generate the protein profile for AML cell line–derived EVs on their own. However, the TMT approach improves the ability of researchers to compare different samples under different conditions when they are collected and analyzed together. Therefore, the TMT quantitative analysis approach can reduce variation between samples and allow for precise quantification between groups. In addition, each experiment was reproducibly identified and a statistical analysis of the list of proteins and quantification values is possible. Third, it would be better to compare the effects of enriched proteins in EVs derived from AML cells on the RFS as opposed to the pre-existing cytogenetic and molecular risks of AML. However, we could not confirm this because of the small sample size in this study. Fourth, this study validated the use of AML-derived EVs from BM blood as a biomarker for determining the treatment direction, but it is necessary to examine whether the same findings would be obtained using other bodily fluids, including peripheral blood. Fifth, it is not certain whether the upregulated proteins in AML cell–derived EVs simply mirror the original AML cells or whether they are enriched in EVs depending on the dynamics of the disease in this study alone. Further research is needed.

This study showed that AML cell–derived EVs could be used to identify biomarkers to predict the survival and therapeutic response and to determine future therapeutic directions for patients when their protein profile is analyzed by MS. In conclusion, AML cell–derived EVs represent key protein cargo targets that carry important protein information from their parental AML cells. Thus, EVs derived from AML can be used as representatives of original AML cells and might be useful to investigate the biology of AML and determine the clinical value of certain observations.

## Data availability

Mass spectrometry global proteomics data sets are available via ProteomeXchange with identifiers, PXD022758 and 10.6019/PXD022758. Information on peptides and proteins identified using the LC-MS/MS data can be found in supplemental Table S1.

## Conflict of interest

Authors declare no competing interests.
