# Supplementary material for: A Proteomic Approach to Understand the Clinical Significance of Acute Myeloid Leukemia–Derived Extracellular Vesicles Reflecting Essential Characteristics of Leukemia
Source: Mol Cell Proteomics. 2020 Dec 8;20:100017. doi: 10.1074/mcp.RA120.002169 (PMC7949255; doi:10.1074/mcp.RA120.002169)
Supplement: Supplemental Table S2 [file mmc2.pdf]

Supplemental Table S2. Functional node activity of kinases in AML cell line-derived EVs

Protein	Accession number	Gene symbol	Fold change (AML/Normal)	ANOVA *p*-value
Tyrosine-protein kinase Lyn	P07948	LYN	4.626	<0.01
MOB kinase activator 3A	Q96BX8	MOB3A	3.712	0.038
Nucleoside diphosphate kinase A	P15531	NME1	3.684	0.023
Receptor of activated protein C kinase 1	P63244	RACK1	3.219	<0.01
DNA-dependent protein kinase catalytic subunit	P78527	PRKDC	3.178	<0.01
Serine/threonine-protein kinase 26	Q9P289	STK26	3.155	0.288
Casein kinase II subunit alpha	P68400	CSNK2A1	2.979	0.078
Activated CDC42 kinase 1	Q07912	TNK2	2.909	<0.01
Tyrosine-protein kinase CSK	P41240	CSK	2.881	**0.049**
Cyclin-dependent kinase 1	P06493	CDK1	2.789	0.15
Nucleoside diphosphate kinase B	P22392	NME2	2.583	<0.01
Serine/threonine-protein kinase N1	Q16512	PKN1	2.444	0.262
Serine/threonine-protein kinase 10	O94804	STK10	2.392	<0.01
Tyrosine-protein kinase SYK	P43405	SYK	2.347	0.015
Mitogen-activated protein kinase kinase kinase kinase 4	O95819	MAP4K4	2.271	0.011
Serine/threonine-protein kinase TAO3	Q9H2K8	TAOK3	2.155	0.143
Creatine kinase M-type	P06732	CKM	2.151	0.203
Creatine kinase B-type	P12277	CKB	2.018	<0.01
Casein kinase II subunit beta	P67870	CSNK2B	1.978	0.011
Casein kinase I isoform gamma-3	Q9Y6M4	CSNK1G3	1.938	0.645
Serine/threonine-protein kinase PAK 2	Q13177	PAK2	1.884	0.239
Protein-tyrosine kinase 2-beta	Q14289	PTK2B	1.839	<0.01
Phosphatidylinositol 4-kinase alpha	P42356	PI4KA	1.759	0.199
MOB kinase activator 1B	Q7L9L4	MOB1B	1.699	0.343
Protein kinase C-binding protein NELL2	Q99435	NELL2	1.607	0.538
TRAF2 and NCK-interacting protein kinase	Q9UKE5	TNIK	1.589	<0.01
Adenosine kinase	P55263	ADK	1.577	<0.01
Serine-threonine kinase receptor-associated protein	Q9Y3F4	STRAP	1.571	0.47
Phosphoglycerate kinase 1	P00558	PGK1	1.547	0.125
Tyrosine-protein kinase Yes	P07947	YES1	1.536	<0.01
Golgi-associated kinase 1B	Q6UWH4	GASK1B	1.392	0.593
cAMP-dependent protein kinase type I-alpha regulatory subunit	P10644	PRKAR1A	1.36	0.062
Ribosomal protein S6 kinase alpha-3	P51812	RPS6KA3	1.337	0.287
Serine/threonine-protein kinase MRCK beta	Q9Y5S2	CDC42BPB	1.331	0.743
Rho-associated protein kinase 1	Q13464	ROCK1	1.256	0.083
cAMP-dependent protein kinase catalytic subunit alpha	P17612	PRKACA	1.255	0.955

Triokinase/FMN cyclase	Q3LXA3	TKFC	1.222	0.988
Bifunctional UDP-N-acetylglucosamine 2-epimerase/N-acetylmannosamine kinase	Q9Y223	GNE	1.133	0.606
Dual specificity mitogen-activated protein kinase kinase 2	P36507	MAP2K2	1.05	0.981
Hepatocyte growth factor-regulated tyrosine kinase substrate	O14964	HGS	1.048	0.133
Pyruvate kinase PKM	P14618	PKM	1.037	0.184
Mitogen-activated protein kinase 1	P28482	MAPK1	0.998	0.8
ATP-dependent 6-phosphofructokinase, platelet type	Q01813	PFKP	0.992	0.929
Phosphatidylinositol 5-phosphate 4-kinase type-2 alpha	P48426	PIP4K2A	0.977	0.868
Myosin light chain kinase, smooth muscle	Q15746	MYLK	0.947	0.982
Kinase D-interacting substrate of 220 kDa	Q9ULH0	KIDINS220	0.943	0.381
cAMP-dependent protein kinase type II-beta regulatory subunit	P31323	PRKAR2B	0.843	0.671
Inactive tyrosine-protein kinase 7	Q13308	PTK7	0.779	0.6
Pyruvate kinase PKLR	P30613	PKLR	0.751	0.385
Extracellular serine/threonine protein kinase FAM20C	Q8IXL6	FAM20C	0.71	0.138
Protein kinase C beta type	P05771	PRKCB	0.682	0.369
cAMP-dependent protein kinase type II-alpha regulatory subunit	P13861	PRKAR2A	0.682	0.831
Adenylate kinase isoenzyme 1	P00568	AK1	0.644	0.05
Integrin-linked protein kinase	Q13418	ILK	0.636	0.024
Galactokinase	P51570	GALK1	0.625	0.023
Myristoylated alanine-rich C-kinase substrate	P29966	MARCKS	0.146	0.231
